# E-cigarettes and heated tobacco products impact on dental color parameters^☆^

**DOI:** 10.1016/j.heliyon.2024.e24084

**Published:** 2024-01-26

**Authors:** Shipra Gupta, Vaibhav Sahni, Rosalia Emma, Stefan Gospodaru, Gheorghe Bordeniuc, Valeriu Fala, Amaliya Amaliya, Giusy Rita Maria La Rosa, Sebastiano Antonio Pacino, Salvatore Urso, Hasan Guney Yilmaz, Giovanni Zucchelli, Riccardo Polosa

**Affiliations:** a Unit of Periodontics, Oral Health Sciences Centre, Post Graduate Institute of Medical Education and Research (PGIMER), Chandigarh, India; b Department of Biomedical and Biotechnological Sciences, University of Catania, Italy; c Faladental, Chișinău, Republic of Moldova; d “Nicolae Testemiţanu” State University of Medicine and Pharmacy, Chişinău, Republic of Moldova; e Department of Periodontology, Faculty of Dentistry, Universitas Padjadjaran, West Java, Indonesia; f ECLAT Srl, Spin-off of the University of Catania, Catania, Italy; g Addendo Srl, Dental Clinic, Catania, Italy; h Department of Biological, Geological and Environmental Sciences, University of Catania, Catania, Italy; i Near East University, Faculty of Dentistry. Department of Periodontology. Nicosia, Mersin10, Turkey; j Department of Biomedical and Neuromotor Sciences, University of Bologna, Bologna, Italy; k Center of Excellence for the Acceleration of HArm Reduction (CoEHAR), University of Catania, Italy; l Department of Clinical and Experimental Medicine, University of Catania, Catania, Italy; m Centre for the Prevention and Treatment of Tobacco Addiction (CPCT), Teaching Hospital, Catania, Italy; n Policlinico Universitario - V. Emanuele", University of Catania, Catania, Italy

**Keywords:** Dental color, Digital spectrophotometer, Smoking, e-cigarettes, Heated tobacco products

## Abstract

**Objectives:**

Abstaining from tobacco smoking may not only improve general health, but also reduce teeth staining and restore teeth whiteness. Compared with conventional cigarettes, E-cigarettes (ECs) and heated tobacco products (HTPs) may offer substantial reduction in exposure to pigmented tar-like compounds of cigarette smoke. It is possible that improvements in dental color indices may be observed in those who have stopped smoking combustible cigarettes by switching to tar-free nicotine delivery products.

**Methods:**

This cross-sectional study evaluated and compared dental color parameters by digital spectrophotometry among five different groups: individuals who currently smoke ; individuals who used to smoke but have quit ; individuals who have never smoked ; exclusive users of electronic cigarettes (former smokers) ; and exclusive users of heated tobacco products (former smokers) .

**Results:**

Dental whiteness in current cigarette smokers was notably worse compared with never and former smokers, (13.38 Whiteness Index for Dentistry (WID) units vs. 19.96 and 16.79 WID units). Remarkably high WID values (i.e., whiter teeth) were also observed in ECs (16.72 WID units) and HTPs users (17.82 WID units). Compared to current smokers, difference in dental whiteness for ECs and HTPs users was visually noticeable (ΔWID difference being on average > 2.90 units). The colour differences measured as delta E*(ΔE*) were all visually detectable except for the comparison between ex-smokers and ECs users for which no perceptible color difference was observed (0.415).

**Conclusion:**

Exclusive use of ECs and HTPs is associated with better dental color measurements than current smoking, suggesting that tar-free nicotine delivery technologies are unlikely to have negative effects on dental appearance.

**Clinical significance:**

Use of alternative nicotine delivery systems may be associated with cosmetic benefits with important implications for those smokers perceiving dental aesthetics as a significant problem. For these an oral-based narrative may be a much more significant reason to refrain from smoking than the fear of developing smoking-related diseases in future.

## 1 Introduction

Tobacco smoking is common with 1.1 billion smokers in 2019 worldwide [[1], [2], [3]]. In addition to being the main cause of lung cancer, chronic obstructive pulmonary disease, and cardiovascular disease [2] tobacco smoking is also known to contribute to poor oral health and tooth discoloration [4,5].

Chronic inhalation of combustion chemicals in tobacco cigarette smoke consists of pigments that can cause progressive teeth staining (ranging from yellow to dark brown or gray in color) and discoloration [6], the intensity being strongly associated with the frequency and duration of cigarette smoking [7,8].

Choosing not to smoke tobacco not only improves overall health but also helps reduce tooth staining and restore the natural whiteness of teeth. Recent observational studies have highlighted that individuals who do not smoke exhibit significantly better tooth whiteness compared to smokers [9]. These findings suggest that the dental appearance of smokers can improve once they cease exposure to the harmful chemicals found in cigarette smoke. Thus, measuring dental color parameters can serve as a valuable indicator for assessing dental aesthetics in studies focused on smoking cessation and the use of tar-free nicotine delivery alternatives such as e-cigarettes, heated tobacco products, and oral tobacco/nicotine products.

E-cigarettes (ECs) and heated tobacco products (HTPs) have evolved as popular, yet controversial, tobacco cigarettes substitute among smokers worldwide [[10], [11], [12], [13]]. Compared to conventional cigarettes, they offer substantial reduction in exposure to toxic chemical emissions [[14], [15], [16], [17], [18], [19]] and, for this reason, they are proposed for harm reduction from cigarette smoke [20,21] and for smoking cessation [22,23].

Switching to exclusive use of ECs and HTPs may not only reduce harm from cigarette smoke, but also the negative aesthetic impact of pigmented tar-like compounds onto the tooth surface. In experimental studies of human premolars extracted for orthodontic reasons [7] and bovine enamel blocks [8], exposure to aerosols generated by ECs and HTPs has been shown to induce little or no dental discoloration [24].

Our hypothesis suggests that a noticeable enhancement in dental color indices can be observed among individuals who have transitioned from smoking conventional tobacco cigarettes to tar-free nicotine delivery products. To examine this hypothesis, we employed digital spectrophotometry, a non-invasive and straightforward method for assessing teeth whiteness, within a cohort exclusively using e-cigarettes and heated tobacco products. Subsequently, we compared the test outcomes with the dental color parameters of current, former, and never smokers, ensuring age and sex matching, based on previous research [9].

The primary aim of this study is to demonstrate whether individuals who have transitioned from smoking tobacco cigarettes to tar-free nicotine delivery products (specifically, exclusive e-cigarette and heated tobacco product users) exhibit improved dental color indices compared to regular tobacco smokers. The secondary objective involves exploring potential interactions among various covariates—such as age, gender, and tooth brushing frequency—on the dental color indices among the studied groups.

## 2 Materials and methods

Several aspects of this section have already been described in our previous publication [9].

The principal investigator and study team adhered to the STROBE guidelines throughout the planning, execution, and reporting phases of the study. All the measures regarding study design, participant selection, data collection, data and statistical analysis, limitations, ethics approval, results, were implemented to comply with the STROBE protocol.

## 3 Study population

The study includes five distinct study groups. Smokers and former smokers were selected from a group of individuals who had visited a smoking cessation clinic (CPCT, Centro per la Prevenzione e Cura del Tabagismo of the University of Catania) within the past two years. Never smokers, as well as users of ECs and HTPs, were recruited from hospital staff, university students, local dental clinics, and through social media. The study participants were categorized as follows.

1. Current smokers: smokers of at least 10 cigarettes per day, with an exhaled carbon monoxide (eCO) level ≥7 ppm;
2. Former smokers: smokers who had completely stopped smoking ≥6 months, and with an eCO level <7 ppm;
3. Never smokers: people who reported having smoked less than 100 cigarettes in their lifetime, and with an eCO level <7 ppm (to exclude subjects significantly exposed to environmental cigarette smoke);
4. ECs users: former smokers who had completely stopped smoking ≥6 months after switching to EC use, and with an eCO level of <7 ppm;
5. HTPs users: former smokers who had completely stopped smoking ≥6 months after switching to HTPs use, and with an eCO level of <7 ppm.

Subjects in the study population had to satisfy the following inclusion and exclusion criteria:

Inclusion criteria - a) Healthy adult subjects (18–50 years of age). b) Presence of at least ten natural anterior teeth (from cuspid to cuspid) in both the upper and lower jaws, without any composite restorations or prosthetics/crowns.

## 4 Exclusion criteria

a) Conditions that might influence dental color measurements, such as daily use of mouthwashing containing chlorhexidine, cetylpyridinium chloride, or essential oils for at least 7 days prior to screening.
b) Subjects wearing removable or fixed orthodontic appliances or prostheses (restricted only to the 12 natural anterior teeth).
c) Significant exposure to environmental tobacco smoke (excluding current smokers).
d) Professional dental hygiene within the past 24 weeks prior to screening.
e) Pregnancy.

The study adhered to the Declaration of Helsinki and the Principles of Good Clinical Practice.

## 5 Study design

This cross-sectional study aimed to measure and compare digital spectrophotometric parameters for dental color among five study populations: (1) current smokers, (2) former smokers, (3) never smokers, (4) exclusive users of ECs who were former smokers, and (5) exclusive users of HTPs who were former smokers. The study groups were carefully matched for age and gender using a dedicated macro in the SAS software.

During the screening process, potential participants were provided with information regarding the purpose and objectives of the research. They underwent eligibility criteria screening and were evaluated for their nicotine usage (including tobacco cigarette consumption, use of ECs, and HTPs) as well as their oral hygiene habits (such as tooth brushing frequency and type of toothpaste used). Basic socio-demographic characteristics including gender, age, and occupation, were also recorded.

Eligible participants then attended a study session for dental color measurements using digital spectrophotometry. Participants were instructed to.

1) Maintain their usual oral hygiene routine (tooth brushing, mouth washing, interdental flossing).
2) Avoid scaling and polishing procedures.
3) Abstain from smoking for at least 2 h prior to the study session.
4) Refrain from brushing their teeth for at least 2 h before the study session.
5) Abstain from eating and drinking (except water) for at least 2 h before the study session.

During the study session, after confirming eligibility criteria and reviewing study instructions, measurements of eCO and dental color assessment were conducted, and the data were recorded.

### 5.1 Exhaled carbon monoxide (eCO) measurement

The smoking status was confirmed objectively by assessing eCO levels (>7 parts per million [ppm], indicative of smoking status) using a handheld CO monitor (Micro CO; Micro Medical Ltd, UK). Participants were requested to abstain from smoking cigarettes for a minimum of 2 h before the eCO measurements. They were instructed to exhale slowly into a disposable mouthpiece connected to the eCO monitor in accordance with the manufacturer's guidelines. The recorded eCO readings were duly noted.

### 5.2 Dental color assessment

Before the dental color assessment, subjects were asked to rinse their mouths with water.

Subsequently, gentle flushing and drying with a triple syringe were conducted to ensure a clean surface, removing any food debris.

All measurements were consistently performed in the same examination room, maintaining identical ambient lighting conditions and executed by the same operator. The digital spectrophotometer (Vita Easyshade V) was calibrated and utilized following the manufacturer's recommendations., Participants were instructed to open their mouths with positioning their tongues away from the anterior teeth.

The color measurement was focused on the central tooth area of the outer surface using the modality "base shade determination". To ensure accuracy, the measuring tip was held at a 90° angle to the tooth surface to ensure precise measurements. Participants were requested to briefly hold their breath during the measurement to prevent fogging of the measuring tip, which could compromise accuracy of color readings.

CIE L*a*b* color parameters were recorded for the vestibular aspect of each anterior tooth (from cuspid to cuspid, upper and lower jaw).

These parameters include L* measuring lightness (ranging from 0 for black to 100 for white), while the a* and b* express chromaticity measures for green/red and blue/yellow, respectively. The total CIE L*a*b* scores for each subject were obtained by summing the individual values of all examined anterior teeth and dividing by the number of teeth examined.

### 5.3 Whiteness index for dentistry (WID)

The WID, derived from CIE L*a*b* coordinates, was calculated using the equation [25]:

WID = 0.511L∗ −2.324a∗ −1.100b∗

Each participant's total WID values were obtained by summing the values of all tested anterior teeth and dividing by the number of teeth examined. Higher WID values indicate whiter teeth, while lower WID values suggest discoloration or less white teeth. Differences in the WID index were assessed in relation to the whiteness 50:50 % acceptability threshold (WAT = 2.90 ΔWID units) [26]. A ΔWID difference >2.90 units suggests a visible variation in whiteness between two teeth.

### 5.4 Delta E* calculation for color variation assessment

The calculation of the color variation delta E* (ΔE*) between the mean values of each study group was calculated as follows [24]:

ΔE*=[(L1*−L2*)2+(a1*−a2*)2+(b1*−b2*)2]12

Values ranging from 0.0 to 1.1 were considered as not perceptible, between 1.1 and 3.3 as visually perceptible, but clinically acceptable, while all ΔE* higher than 3.3 were considered as clearly visible and clinically disturbing [27].

### 5.5 Data analysis

To evaluate the normal distribution of the data, the Kolmogorov-Smirnov test was utilized. Categorical data were presented as counts and percentages, while continuously distributed data that exhibited a normal distribution were reported using the mean (standard deviation; SD). Continuously distributed data displaying skewness were reported using the median (interquartile range; IQR).

Comparisons of clinical data among the study groups were performed using the Chi-square test for categorical data, one-way ANOVA for normally distributed continuous data, and the Kruskal-Wallis test for skewed continuous data. Regression analyses were performed for each dental color parameter including study groups (smokers, ex-smokers, never smokers, ECs users, and HTPs users) age, gender, eCO levels, toothbrushing frequency (daily), mouth washing frequency (daily), and dental flossing frequency (weekly) to assess the interactions. Following the results obtained from each regression model, comparisons among study groups were performed by applying ANCOVA (using type III errors) with study groups, age, gender, and toothbrushing frequency (daily) as covariates. The Tukey's post-hoc analysis was applied for multiple comparisons. All analyses were considered significant with a P value < 0.05. R version 3.4.3 (2017-11-30) was utilized for data analysis and generation of graphs.

## 6 Results

### 6.1 Study participants

A total of 89 subjects (mean ± SD age of 34.39 ± 10.4 years; 34 Female) were recruited in this study, including 18 smokers, 18 ex-smokers, 20 never smokers, 15 HTPs users, and 18 ECs users. All the characteristics of study groups were reported in Table 1. As anticipated, considerable differences were noted in eCO levels (p < 0.0001) among the study groups.

**Table 1:** Clinical characteristic of study groups.

	Smokers	Ex- Smokers	Never Smokers	HTPs users	ECs users	*P* value
Subjects n.	18	18	20	15	18	
Age (yr)	34 ± 10.3	35.78 ± 12.7	34.04 ± 7.6	32.27 ± 9.2	35.56 ± 12.3	0.881
Female	4/18 (22.2 %)	7/18 (38.9 %)	10/20 (50 %)	6/15 (40 %)	7/18 (38.9 %)	0.532
Exhaled CO	14.5 (11–17.3)	3 (3–4)	2.5 (2–4)	2 (2–3)	2.5 (2–3)	**<0.0001**
Toothbrushing frequency (daily)	2 (1.4–3)	2 (2–3)	2 (2–2.4)	2 (2–2)	2 (2–3)	0.775
Mouth washing frequency (daily)	0 (0–0)	0 (0–0.3)	0 (0–2)	0 (0–1)	0 (0–0.3)	0.060
Dental flossing frequency (weekly)	0 (0–1.9)	1 (0–1.5)	0.5 (0–1.5)	1 (0–1)	0 (0–1.5)	0.883
N. Cig./Day	15 (12–19.5)	//	//	//	//	NA
Year smoking	11 (8.5–19.75)	//	//	//	//	NA
Pack/years	9.75 (5.6–12.5)	//	//	//	//	NA
Year non-smoking	//	2.5 (1.5–7.65)	//	2 (1.5–2)	2 (2–4.3)	0.263
ECs type refillable/others	//	//	//	//	15/3	NA
HTPs type IQOS/glo	//	//	//	13/2	//	NA

Data are summarized as mean ± standard deviation (SD), median (IQR), or n/N (%) unless otherwise stated. NA: not applicable.

However, no significant differences were detected in any of the other parameters.

### 6.2 Assessment of interaction effects on dental color parameters

The influence on dental color measurements was assessed for different parameters, including study groups (current smokers, former smokers, never smokers, ECs users, and HTPs users) age, gender, eCO levels, toothbrushing frequency (daily), mouth washing frequency (daily), and dental flossing frequency (weekly).

The covariate “Age” was significantly related to L* (p < 0.0001), a* (p = 0.0004), and WID (p = 0.016) values. The covariate “Sex” was significantly related to a* (p = 0.023), b* (p = 0.0006), and WID (p = 0.007) values. The covariate “toothbrushing frequency (daily)” was significantly related to a* (p = 0.001), b* (p = 0.003), and WID (p = 0.004) values. No significant effect was observed for the other covariates.

### 6.3 Comparisons of dental color parameters among study groups

**Fig. 1:**
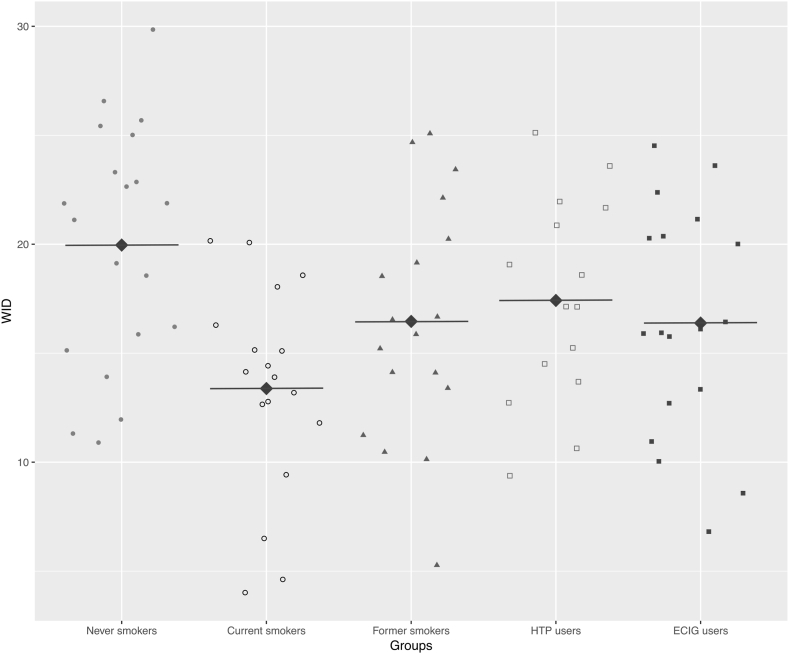
Comparison of WID units among smokers, ex-smokers, never smokers, HTPs users, and ECs users' groups. Each dot represents the individual values of WID measurements. The rhombus dots represent the mean of WID units for each study group.

Overall, results of comparisons among groups for dental color parameters were summarized in Table 2. Multiple comparisons for L*, a*, b*, and WID parameters were showed respectively in Table 3, Table 4, Table 5, Table 6. No significant difference was observed for the L* parameter among the study groups (p = 0.227). Moreover, L* parameter showed no significant differences when multiple comparisons were performed (p values > 0.05). Similar results were observed for the a* parameter, which showed no significant difference either among the study groups (p = 0.175) and when multiple comparisons were performed (p values > 0.05). Instead, significant differences were observed for both b* (p = 0.0009) and WID (p = 0.017) between study groups. Also, multiple comparisons showed significant differences between smokers and never smokers for both b* (p = 0.001) and WID (p = 0.005) (Fig. 1). No other differences were observed for the other multiple comparisons (see Table 4, Table 5, Table 6).

**Table 2:** Dental color comparisons among smokers, ex-smokers, never smokers, HTPs users, and ECs users.

	Smokers	Ex- Smokers	Never Smokers	HTPs users	ECs users	*P* value
L*	79.77 (73.7–81.57)	79.88 (77.1–83.5)	79.79 (76.9–83.3)	80.67 (77.9–83.7)	80.1 (75.5–83.1)	0.227
a*	0.57 ± 0.8	0.29 ± 0.97	−0.17 ± 0.7	0.05 ± 0.8	0.24 ± 1.1	0.175
b*	22.82 ± 2.8	21.01 ± 2.9	18.99 ± 3	21.23 ± 2.9	21.31 ± 2.8	0.009
WID units	13.38 ± 4.8	16.79 ± 6.1	19.96 ± 5.5	17.82 ± 5.6	16.72 ± 5.9	0.017

*Data are summarized as mean* ± *standard deviation (SD) or median (interquartile range - IQR). P values were calculated by applying Tukey's post-hoc test*.

**Table 3:** Adjusted p values for L* comparisons among smokers, ex-smokers, never smokers, HTPs users, and ECs users.

	Smokers	Ex-Smokers	Never Smokers	HTPs users	ECs users
Smokers		0.841	0.903	0.335	0.743
Ex-Smokers	0.841		0.999	0.896	0.999
Never Smokers	0.903	0.999		0.812	0.996
HTPs users	0.335	0.896	0.812		0.950
ECs users	0.743	0.999	0.996	0.950	

*P* values were calculated by applying Tukey's post-hoc test.

**Table 4:** Adjusted p values for a* comparisons among smokers, ex-smokers, never smokers, HTPs users, and ECs users.

	Smokers	Ex-Smokers	Never Smokers	HTPs users	ECs users
**Smokers**		0.872	0.072	0.433	0.783
**Ex-Smokers**	0.872		0.465	0.931	0.999
**Never Smokers**	0.072	0.465		0.940	0.583
**HTPs users**	0.433	0.931	0.940		0.971
**ECs users**	0.783	0.999	0.583	0.971	

*P* values were calculated by applying Tukey's post-hoc test.

**Table 5:** Adjusted p values for b* comparisons among smokers, ex-smokers, never smokers, HTPs users, and ECs users.

	Smokers	Ex-Smokers	Never Smokers	HTPs users	ECs users
Smokers		0.331	**0.001**	0.512	0.521
Ex-Smokers	0.331		0.206	0.999	0.998
Never Smokers	**0.001**	0.206		0.163	0.104
HTPs users	0.512	0.999	0.163		0.999
ECs users	0.521	0.998	0.104	0.999	

*P* values were calculated by applying Tukey's post-hoc test.

**Table 6:** Adjusted p values for WID comparisons among smokers, ex-smokers, never smokers, HTPs users, and ECs users.

	Smokers	Ex-Smokers	Never Smokers	HTPs users	ECs users
Smokers		0.366	**0.005**	0.166	0.389
Ex-Smokers	0.366		0.416	0.985	0.999
Never Smokers	**0.005**	0.416		0.797	0.391
HTPs users	0.166	0.985	0.797		0.980
ECs users	0.389	0.999	0.391	0.980	

*P* values were calculated by applying Tukey's post-hoc test.

The variation in color was assessed using the ΔE* parameter calculated between the mean of L*, a*, and b* of the study groups (Table 7). The ΔE* values for smokers vs never smokers (4.102) and smokers vs HTPs users (3.366) showed a clearly visible color difference with ΔE* values higher than 3.3. Visually perceptible color differences were observed for smokers vs ex-smokers (2.384), smokers vs ECs users (2.366), ex-smokers vs never smokers, ex-smokers vs HTPs users, never smokers vs HTPs users, never smokers vs ECs users, with all ΔE* values between 1.1 and 3.3. No perceptible color difference was observed for ex-smokers vs ECs users (0.415).

**Table 7:** ΔE* evaluation among study groups.

	Smokers	Ex-Smokers	Never Smokers	HTPs users	ECs users
Smokers		2.384	4.102	3.366	2.366
Ex-Smokers			2.084	1.435	0.415
Never Smokers				2.789	2.418
HTPs users					1.144
ECs users					

## 7 Discussion

When compared to tobacco cigarettes, ECs and HTPs potentially provide significant reductions in exposure to pigmented tar-like substances found in cigarette smoke. Nevertheless, there is limited knowledge regarding the impact of aerosol emissions from tar-free nicotine delivery devices on dental color. This study is the first to examine dental color among users of ECs and HTPs.

Dental whiteness in current cigarette smokers was notably worse compared with never and former smokers, (13.38 WID units vs. 19.96 and 16.79 WID units). Remarkably high WID values (i.e., whiter teeth) were also observed in ECs (16.72 WID units) and HTPs users (17.82 WID units). Compared to current smokers, the difference in dental whiteness for ECs and HTPs users was visually noticeable (ΔWID difference being on average > 2.90 units). This is a novel finding that is due to products’ design that does not require combustion to operate thus avoiding the production of tar-associated pigments of tobacco smoke.

In the present study, the ΔE* was used to analyze color variation across different study groups [28]. Notable, smokers compared with never smokers and smokers compared with HTPs users showed visible and clinically impacting differences in color, while the other group comparisons exhibited ΔE* values between 1.1 and 3.3 which correspond to differences visually detectable but clinically acceptable. Interestingly, ex-smokers compared to ECs users showed no discernible differences in color. These findings suggest that traditional cigarettes exhibit a more pronounced effect in color parameters, potentially due to their chemical composition. On the other hand, HTPs and ECs seem to have a milder influence on dental color indices due to the reduced chemical emissions compared to traditional cigarettes.

The similarity in dental color parameters between exclusive EC and HTPs users and ex- or never smokers aligns with existing knowledge regarding tar-associated pigments in tobacco smoke. It is well-known that smokers tend to experience greater dental discoloration in comparison to non-smokers [[6], [7], [8],^29^]. Consequently, it is plausible that quitting smoking would result in a visible improvement in dental color.

Taking into account that exclusive users of ECs and HTPs in our study have, on average, abstained from smoking for only 2 years, the reported improvement in dental color indexes may occur within a relatively short period of time after transitioning to tar-free devices following the cessation of tobacco cigarette use. However, prospective studies are needed to determine the precise timeline of changes in dental color after quitting smoking.

The visually noticeable difference in dental whiteness among exclusive ECs and HTPs users compared to regular smokers is likely due to substantial reduction of tooth surface exposure to tar-associated pigments of tobacco smoke. The role of tar in causing tooth discoloration is expected; as an example, the staining intensity of cigarette filters is directly proportional to tar production [30,31]. Moreover, experimental studies showing that exposure to aerosols generated by ECs and HTPs is responsible for a lower degree of dental discoloration compared to cigarette smoke are in support of our findings [6,7,32,33].

Age, sex and toothbrushing frequency were among the individual variables that significantly influenced dental color measurements. Age and gender are well known determinants of the natural color of the teeth. Increasing age tends to be associated with darker teeth which are more yellow in their hue and women tend to exhibit lighter as well as less chromatic shades of the dentition than men [[34], [35], [36], [37]].

Personal oral hygiene may also influence dental color appearance [[38], [39], [40]]. Our multiple regression analyses showed that tooth brushing frequency can act as an additional confounder for dental color measurements.

The role of these covariates could have affected dental color measurements in the study, but subjects’ populations were well matched with no significant differences in term of age, gender and tooth brushing frequency distribution among the five study groups.

The study has a few limitations. First, it is important to note that this is a small exploratory study conducted to build confidence in the usefulness of digital spectrophotometry in clinical research, including an ongoing prospective multicenter randomized controlled trial [41]. Although no human data on WID values were available for power calculation, the substantial dental color difference reported in experimental studies between combustible and non-combustible exposures [7,8] justified the use of a small sample size for this study. The findings of the present study seem to confirm this; an aesthetically relevant difference in WID scores of >2.9 units (the visually perceivable difference in whiteness between two teeth) was consistently observed when comparing parameters of ECs and HTPs users with those of current smokers. Moreover, the relatively small standard deviation and interquartile range suggest minimal measurement variability, implying that a small group of 15–20 subjects can provide informative data. Nonetheless, the absence of significant differences between EC and HTPs users could have resulted from the small sample size. Second, it is acknowledged that the cross-sectional design of this study cannot establish a causal relationship. Third, findings were restricted to selected populations of relatively young participants, thus limiting the generalizability of their dental color findings. Consequently, confirmatory studies with more representative age groups are needed. Fourth, although we took careful consideration of many key modulators of dental color, we cannot exclude the possibility of possible residual confounding factors that were not assessed. For example, careful assessment of red wine and coffee consumption, which are known to significantly stain dental surfaces [42,43], was not included. However, participants were instructed to avoid consumption of red wine and coffee at least 2 h before study assessments. A thorough standardized approach must be in place when conducting oral health studies.

Lastly, the study was conducted between the first and second waves of the pandemic in Italy, during a period when clear guidelines for dental settings were already established. As a result, COVID-19 restrictions had only a minimal impact on the study's execution.

Considering the limitations of the study, it can be reasonably argued that the exclusive use of alternative nicotine delivery systems such as heated tobacco products and e-cigarettes is linked to improved dental color measurements compared to current smoking. This suggests that tar-free nicotine delivery technologies are unlikely to have detrimental effects on dental appearance. However, these findings need to be confirmed through large-scale, meticulously designed prospective studies.

The potential cosmetic benefits associated with the use of heated tobacco products and e-cigarettes may have significant implications for smokers, especially young smokers who perceive dental aesthetics as a significant concern [29,44]. For these individuals, an oral-centric narrative (such as achieving a healthier and brighter smile) may serve as a more compelling reason to quit smoking than the fear of future lung cancer or cardiopulmonary diseases.

## Funding

Funding for this investigator-initiated study was provided by ECLAT Srl., a spin-off of the 10.13039/501100004505University of Catania, through a grant from the Foundation for a Smoke-Free World. The Foundation for a Smoke-Free World is a US-based nonprofit 501(c)(3) private foundation dedicated to ending smoking in the current generation. The authors are solely responsible for the content, selection, and presentation of facts, as well as any opinions expressed in this study. These views should not be interpreted as reflecting the positions of the Foundation for a Smoke-Free World, Inc. ECLAT, a spin-off company of the University of Catania, focuses on delivering solutions to global health issues, with a particular emphasis on harm reduction and technological innovation.

## Ethical approval

The study was conducted according to the Principles of Good Clinical Practice (GCP) and Declaration of Helsinki. Informed consent was obtained from all individual participants included in the study.

## Clinical trial registration

The study is purely observational, with no assignment of any medical intervention or decisions at the discretion of the investigator, so it not requires registration.

The study is a pilot study parts of a larger project with registration ID: NCT04649645, which was approved by the local ERB (Catania ASP; approval no. 697, dated November 18, 2020). As preliminary study, the pilot study referred into this paper started before the larger study registered in ClinicalTrials.gov.

## Data availability statement

The datasets used and analyzed during the current study are available from the corresponding author on reasonable request.

## CRediT authorship contribution statement

**Shipra Gupta:** Writing – review & editing, Writing – original draft, Validation, Formal analysis, Data curation, Conceptualization. **Vaibhav Sahni:** Writing – review & editing, Writing – original draft, Formal analysis, Conceptualization. **Rosalia Emma:** Writing – review & editing, Writing – original draft, Formal analysis, Data curation, Conceptualization. **Stefan Gospodaru:** Writing – review & editing, Writing – original draft, Validation, Formal analysis, Data curation. **Gheorghe Bordeniuc:** Writing – review & editing, Writing – original draft, Formal analysis, Data curation. **Valeriu Fala:** Writing – review & editing, Writing – original draft, Validation, Formal analysis, Data curation, Conceptualization. **Amaliya Amaliya:** Writing – review & editing, Writing – original draft, Formal analysis, Data curation, Conceptualization. **Giusy Rita Maria La Rosa:** Writing – review & editing, Writing – original draft, Formal analysis, Data curation. **Sebastiano Antonio Pacino:** Writing – review & editing, Writing – original draft, Formal analysis, Data curation, Conceptualization. **Salvatore Urso:** Writing – review & editing, Writing – original draft, Formal analysis, Data curation, Conceptualization. **Hasan Guney Yilmaz:** Writing – review & editing, Writing – original draft, Formal analysis, Data curation, Conceptualization. **Giovanni Zucchelli:** Writing – review & editing, Writing – original draft, Formal analysis, Data curation, Conceptualization. **Riccardo Polosa:** Writing – review & editing, Writing – original draft, Data curation, Conceptualization.

## Declaration of competing interest

The authors declare the following financial interests/personal relationships which may be considered as potential competing interests.
